# The effect of intelligent reflecting surfaces on spectral efficiency in 6G wireless systems

**DOI:** 10.1038/s41598-025-03468-9

**Published:** 2025-06-02

**Authors:** Hanaa H. Qamar, Hesham M. Elbadawy, Abdelhady A. Ammar

**Affiliations:** 1 Electronics and Electrical Communications Department, Faculty of Engineering, Al-Azhar University, Cairo, Egypt; 2 National Telecommunications Institute, Cairo, Egypt; 3 Electronics and Electrical Communications Department, Madina Higher Institute for Engineering and Technology, Giza, Egypt

**Keywords:** Wireless communication, Intelligent reflecting surface, 6G, Signal to noise ratio, Data rate, The spectral efficiency, The energy efficiency, Engineering, Electrical and electronic engineering

## Abstract

Intelligent Reflecting Surfaces (IRSs), comprised of numerous reflecting elements, offer a promising solution for enhancing wireless communication. By manipulating the wireless propagation environment, IRSs can mitigate the effects of non-line-of-sight propagation and extend signal coverage. This paper focuses on the performance of an IRS-aided wireless communication system when the direct link between the Base Station (BS) and receiver is obstructed, relying solely on the IRS for signal transmission, by using the pilot transmission and channel least square method in calculation of the received signal. We investigate the impact of IRS element components (effective capacitance $C$, bottom layer inductance ${L}_{1}$, top layer inductance ${L}_{2}$, and effective resistance R) and the frequency of the incident signal on system performance. These components significantly have an influence on reflection coefficients, where higher values of them leads to an increase in the amplitude response of reflection coefficient and hence, stronger reflected signals. The variation of those components can adjust the phase response of the reflected signal. In this paper, assessment of performance of IRS-aided 6G wireless communication system for single user is studied. The received power, SNR, and the spectral efficiency were estimated and used as metrics for the system performance. It has been cleared that the received power, SNR and the spectral efficiency are increased with increasing the size of IRS and transmitted power for both of the strongest and weakest configuration. Also, it has been shown that the received power, SNR and the spectral efficiency are decreased with increasing the AP-IRS horizontal distance. Higher degree of bandwidth improves the received power and the spectral efficiency whereas it reduces the SNR. The performance comparison between the spectral efficiency of system with IRS and without IRS at different values of transmitted power and bandwidth is introduced. It is shown that, at bandwidth = 5 MHz, the system with IRS achieves nearly 3 times the spectral efficiency of the system without IRS and at bandwidth = 25 MHz, the system with IRS achieves nearly 14 times the spectral efficiency of the system without IRS. Also, at transmitted power = 0.5 Watt, the system with IRS achieves nearly 5 times the spectral efficiency of the system without IRS and at transmitted power = 2.5 Watt, the system with IRS achieves nearly 5.7 times the spectral efficiency of the system without IRS. So, The IRS can be used and aided in improvement of the spectral efficiency in 6G wireless communication. The evaluation of performance of IRS-aided wireless communication system in a multi-user scenario is discussed. The data rate, spectral and energy efficiency were evaluated by using four different methods (power method, uniform metal surface method, IRS with best pilot method and with the case of no IRS). It has been shown that the received power method and the best pilot method give the best data rate, spectral and energy efficiency at any value for each of the transmitted power, the bandwidth, the size of IRS and AP-IRS horizontal distance and consequently, enhance the performance of IRS- aided 6G wireless communication system.

## Introduction

Due to development and research efforts on the optimization and design of the transmitters and receivers, the wireless communication systems performance has improved, for the mobile networks from second generation (2G) to fifth generation (5G). The efficiency of wireless communications has been improved by the improvement of each generation of mobile networks. However, the propagation channel is uncontrollable and sometimes unfavorable to the electromagnetic waves (EM) propagation. This is one of the problems that have been recognized for mobile networks of next generation^1^.

Lately, the technology of IRS has been suggested and researched for implementation to wireless communication systems^2^. Intelligent reflecting surface, commonly referred to as a reconfigurable intelligent surface (RIS) and has multiple alternative titles, such as large intelligent surface (LIS) as mentioned in^3^, large intelligent meta-surface, reconfigurable meta-surface, software-defined surface, software-defined meta-surface. An IRS is a planner antenna array that comprises a lot of passive reflecting components, where each element can smartly reconfigurable phase, amplitude, polarization and frequency of the incident signals and then reflects to the particular receiver to generate constructive interference and enhance the signal^4^. According to this, it is possible to greatly increase wireless communication performance, especially in indoor environments in which signals may be weakened or blocked by obstacles or intensive environments like urban areas. Also, IRS can be configured to drive signals towards required locations or directions not only for improving the quality of signal but also for suppressing undesirable signals which interfere with the wireless communication. Notably, the IRS technology is roughly passive because it is totally based on the EM waves scattering and does not need power amplifiers for transmission of the signals. Actually, some energy is only needed for the intelligent controller that enables the reconfigurability of the IRS. The IRS displays promising possibility for application next generation networks because it can partially monitor and form the wireless propagation channels as one wants. The deployment of IRSs can develop the signal reliability, where it does not require additional intensification for network elements and does not need to use active antennas at both of the transmitters and receivers. IRS technology can be integrated with existing technologies such as millimeter-wave (mmWave) communications, multiple-input multiple-output (MIMO) systems, terahertz (THz) communications, machine learning (ML) and artificial intelligence (AI) for improving the performance of networks of sixth generation (6G). Fortunately, the recent improvements inside (MEMS), or micro-electro-mechanical systems and metamaterials produce the advance of intelligent reflecting surfaces. An overview of research on wireless communication aided by IRS is provided in^5,6^ and a comprehensive analysis of existing channel estimation techniques for IRS is presented in^7^. In^8^, Kaina, et al. first present the concept of LIS to control the wireless communication channel by using tunable surfaces. Furthermore, meta-surfaces are free from radio receiver noise, and don’t need either power amplifiers or analog/digital converters. IRS technology is environmentally friendly because it reduces the overall carbon footprint and consequent to its relative high energy-efficiency^9,10^.

Most of researches on IRS focus on the practical implementation challenges, such as IRS hardware design and energy consumption^11–16^. In^11^ the author studied the design problem of joint active and passive beamformer for systems with multiple inputs and single output (MISO). The effect of discrete phase shift was studied in^12^ and the systems energy consumption was investigated in^13^. In^14^, the hardware implementation of the IRS and the resulting limitations on the practical design of IRS reflection coefficients are discussed. The idea behind IRS’s hardware implementation is "metasurface," which is composed of digitally programmable two-dimensional (2D) metamaterial. The design and applications of an IRS, a 2-dimensional (2D) passive metasurface that can control the wireless propagation channel and consequently, achieve better spectral efficiency and energy efficiency are introduced in^15^. In this paper, the design of IRS consists from three layers where the outer layer is made up of a sizable array of passive reconfigurable patches printed on a dielectric substrate, the intermediate layer reduces signal energy leaks during reflection by using a copper plate and the inner layer makes up of a control circuit board with the capability to steer both of the amplitude and phase reflection in real time. In^16^, the article discuss power consumption and energy efficiency for IRS-aided wireless communication system.

While the aforementioned works have enhanced our knowledge of IRS-assisted communication systems, few works have discussed the analytical performance of IRS assisted systems. With considering that the channel from the transmitter to IRS is deterministic, the authors in^17,18^ studied the MISO systems’ spectral efficiency and outage probability. In^19,20^, the authors assumed that the direct link from transmitter to receiver does not exist, the achievable rate and outage likelihood of a system with a single input and one output (SISO) was presented. In^13^, the authors presented approach for a significant sustainable energy efficient. The authors in^12^ discussed the optimization of beam-forming for wireless communication systems supported by IRS under discrete phase shift restrictions. In^21–26^, is studied maximizing the data rate gains by using several optimization formulations to fully utilize the IRS technology. The secrecy rate is studied in^27,28^ and it is optimized through transmission power optimization of the source and phase shift matrix of IRS. The enhancement of coverage for wireless communication networks deploying IRS is discussed in^29,30^.

In the present work, the performance of IRS-aided wireless communication system when the direct link between the Base BS and receiver is obstructed has been investigated by using the pilot transmission and channel least square method in calculation of the received signal. Firstly, the effect of components of IRS elements and the frequency of the incident signal on reflection coefficients (amplitude and phase response) is investigated. As far as the authors are aware, studying the effect of different values for all components of IRS elements ($RLC$ components) on reflection coefficient and corresponding determine the best values ​​to use in design of elements of IRS that aided 6G wireless communication system has not been documented in the literature yet. In^31^, the authors presented the effect of changing in values of capacitance ($C$) only on the reflection coefficient and in^25^, the authors presented the effect of changing in values both of capacitance ($C$) and resistance ( $R$) only on the reflection coefficient. It is very important to study the effect of different values for all components of IRS elements (capacitance $C$, inductance $L$ and resistance $R$) on reflection coefficient and so, the reflected signal and hence the performance of IRS-aided 6G wireless system. Then, assessment performance of IRS-aided 6G wireless communication system for single user is presented. The received power, SNR, and the spectral efficiency were estimated and used as metrics for the system performance. Analytical performance comparison between the spectral efficiency of system with IRS and without IRS at different values of transmitted power and bandwidth is introduced. Finally, assessment of performance of IRS-aided 6G wireless communication system in a multi-user scenario is presented. The data rate, spectral and energy efficiency were evaluated by using four different methods (power method, uniform metal surface method, IRS with best pilot method and with the case of no IRS) at different values for each of the transmitted power, the bandwidth, the size of IRS and AP-IRS horizontal distance.

## Methodology and system modeling

This section introduces the methodology and work’s formulation of the IRS-aided wireless communication system. First, the generation of intelligent reflecting surface is presented. Next, the description of IRS-aided OFDM communication setup is introduced. Subsequently, the reflection model of the IRS is mathematically formulated. Additionally, the propagation environment and channel modeling are displayed. Finally, the evaluation of spectral and energy efficiency for IRS aided wireless communication are derived by considering the direct path from access point (AP) to user equipments (UEs) is blocked. Specifically, the received signal is calculated by the pilot transmission and channel least square method.

### Generation of intelligent reflecting surface

The numerical assessment of IRS-aided wireless communication systems needs to the implementation of a geometric model to demonstrate the intelligent reflecting surface. The most important properties of such reflecting surface are the reflection properties, operating frequency, and size of surface. To evaluate the reflected signal from such intelligent surface requires the generation of reflecting surface model. In this section, we describe a simple method to generate an intelligent reflecting surface with predetermined statistical properties.

**Fig. 1:**
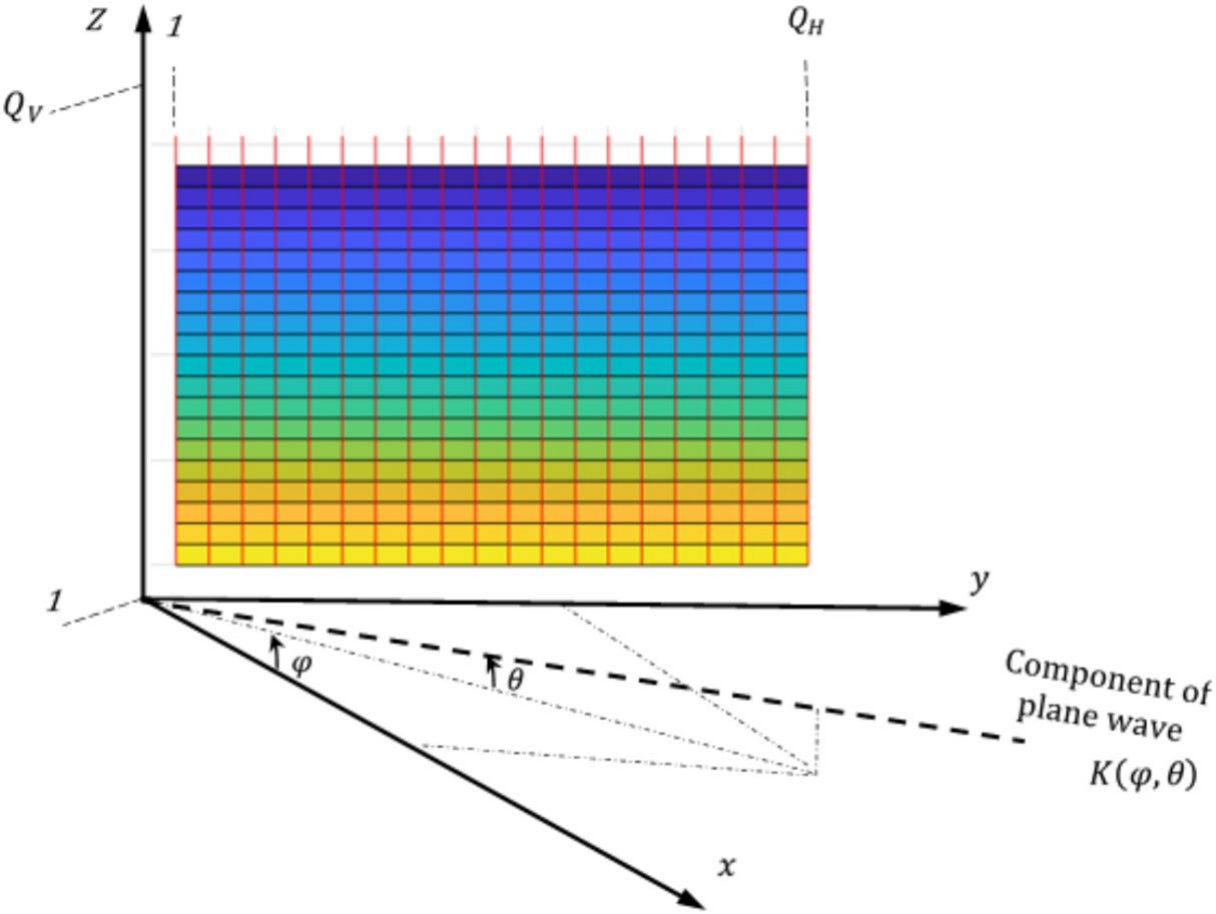
The IRS geometry consisting of uniform planar array with ${Q}_{H}$ components per row and ${Q}_{V}$ components per column.

Let the IRS consists of a uniform planar array with Q = 1024 entries, the elements per horizontal row are ${Q}_{H}=32$ and the elements per vertical column are ${Q}_{V}=32$. The coordinates of each element on the surface are (x, y, z). The elements are uniformly spaced with vertical and horizontal spacing ${l}_{V}={l}_{H}=0.5\lambda$ where we use carrier frequency ${f}_{c}=3.5{\text{ GHz}}$ and bandwidth $B=10{\text{ MHz}}$ for communication during designing of the array. The surface is showed in Fig. 1 and is lied in the y–z plane as in the Cartesian coordinate system described in the figure. Each element on the surface has a directivity pattern or steering vector of $D\left(\varphi ,\theta \right)={cos}^{2}\left(\varphi \right){\text{cos}}\left(\theta \right),$ where $\theta$ is the elevation angle and $\varphi$ is the azimuth angle.

Consider the first element is located at the origin and the elements of reflecting are indexed row-by-row by $q\in \left[1, Q\right],$ hence the nth element location is defined as

1 $${U}_{q}=\left[\begin{array}{c}\begin{array}{c}0\\ i\left(q\right)0.5\lambda \end{array}\\ j\left(q\right)0.5\lambda \end{array}\right]$$

where, $j\left( q \right) = \left\lfloor {\left( {q - 1} \right)/Q_{H} } \right\rfloor { }\;{\text{and}}\;i\left( q \right) = {\text{mod}}\left( {q - 1,Q_{H} } \right)$ are the vertical and horizontal indices of element q, respectively. When a plane wave incident on the IRS, the response vector of array is as in^32^^Sec. 7.3^

2 $$A\left(\varphi ,\theta \right)=\sqrt{D\left(\varphi ,\theta \right)} {\left[{e}^{j{K(\varphi ,\theta )}^{\text{T}}{U}_{1}}, . . . ,{e}^{j{K(\varphi ,\theta )}^{\text{T}}{U}_{Q}} \right]}^{\text{T}}$$

3 $$K\left(\varphi ,\theta \right)=\frac{2\pi }{\lambda } \left[\begin{array}{c}\begin{array}{c}{\text{cos}}\left(\theta \right){\text{cos}}(\varphi )\\ {\text{cos}}(\uptheta )\text{sin}(\varphi )\end{array}\\ sin(\theta )\end{array}\right]$$

where $K\left(\varphi ,\theta \right)$ is the wave vector, the response vector of array calculates the phase shifts between the first element in IRS and the other elements.

### Description of IRS-aided OFDM communication setup

**Fig. 2:**
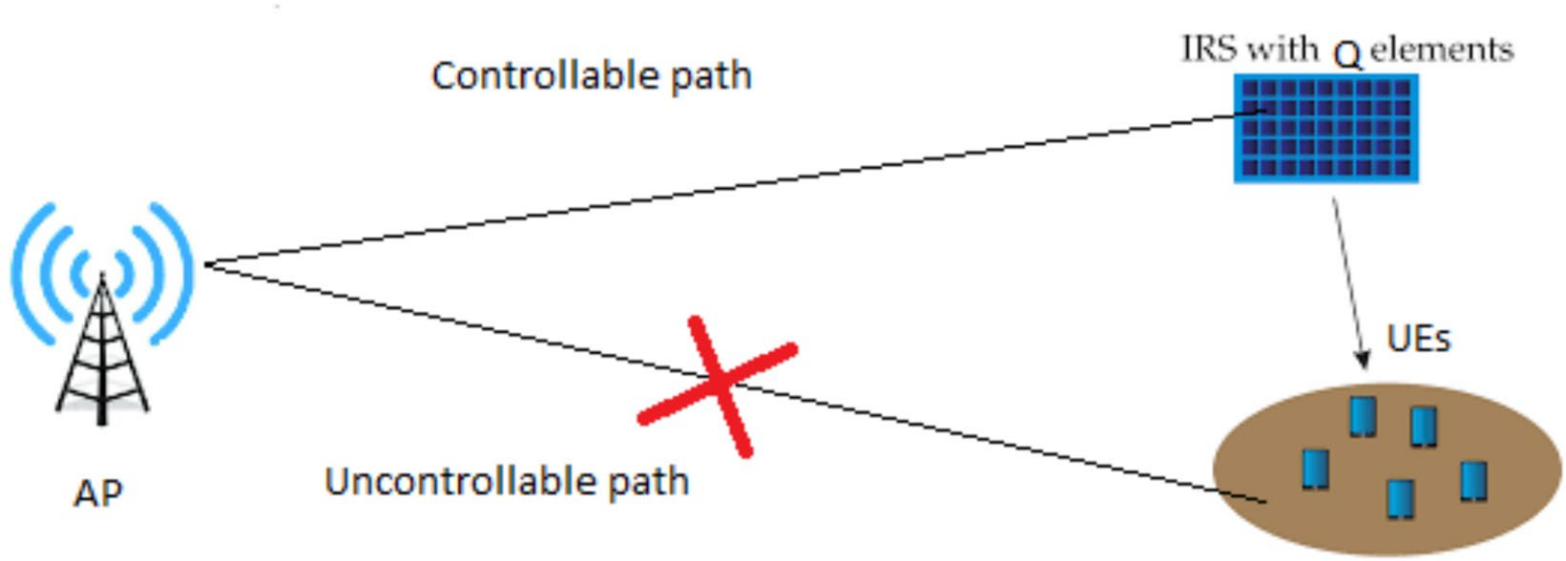
The simulation set up supposes the position of users is next to an IRS. The AP is positioned further away to serve the UEs. There are non-line of sight (NLOS) path from AP to UEs and this path is blocked and line of sight (LOS) path from AP to IRS. The link from IRS to UEs can be LOS or NLOS.

We consider a system where an IRS formed of Q controllable elements is deployed to aid in the communication between the access point (AP) with single antenna and a multitude of user equipments (UEs), as illustrated in Fig. 2. Also, we consider the transmission is executed using the orthogonal frequency division multiple access (OFDM) with pulse shape filter, such as in the model in^33^.

Suppose that the transmitted baseband discrete-time signal in complex domain is donated by x[w], where w is the integer index. The sequence of received discrete-time signal z[w] is defined by

4 $${\varvec{z}}\left[w\right]=\sum_{{\ell}=0}^{N-1}{\mathbf{h}}_{\vartheta }\left[{\ell}\right]\mathbf{x}\left[w-{\ell}\right]+ \mathbf{n}\left[w\right], {\ell}=0,\dots , N-1$$

where, ${\mathbf{h}}_{\vartheta }\left[{\ell}\right]$ is the finite impulse response filter (FIR) which represents broadband channel in the time domain, $\mathbf{n}\left[w\right]$ is the noise of the receiver and N is the taps of FIR filter that depend on the IRS configuration ($\vartheta$) and the physical propagation paths. Tap ${\ell}$ is derived as

5 $${\mathbf{h}}_{\vartheta }\left[{\ell}\right]={\mathbf{h}}_{u}\left[{\ell}\right]+{\mathbf{g}}_{{\ell}}^{\text{T}}{\mathbf{r}}_{\vartheta }$$

where $, {\mathbf{h}}_{u}\left[{\ell}\right]$ is the uncontrollable channel which is equal to zero; when the UEs and BS are directly connected and this link is blocked, ${\mathbf{g}}_{{\ell}}$ is the controllable channel where each element of Q elements of IRS instantly reflect the incident signal and ${\mathbf{r}}_{\vartheta }$ is the IRS’s reflection coefficients where they are the same for all ${\ell}$. Let the B Hz is channel bandwidth or the symbol rate in the communication system, the length of cyclic prefix is N -1 during OFDM transmission and generate $S>N$ subcarriers. Hence, using discrete Fourier transform (DFT), one OFDM block consists of $S$ parallel subcarriers can be generated by the transmission of a block with S + N -1time domain signals:

6 $$\overline{\user2{z}}\left[ m \right] = {\overline{\mathbf{h}}}_{\vartheta } \left[ m \right]{\overline{\mathbf{x}}}\left[ m \right] + {\overline{\mathbf{n}}}\left[ m \right],\quad m = 0, \ldots ,S - {1}$$

where, the DFTs are:

7 $$\overline{{\varvec{z}} }\left[m\right]=\frac{1}{\sqrt{S}}{\sum }_{s=0}^{S-1}\mathbf{z}[s]{e}^{-j2\pi sm/S}$$

8 $$\overline{\mathbf{x} }\left[m\right]=\frac{1}{\sqrt{S}}{\sum }_{s=0}^{S-1}\mathbf{x}[s]{e}^{-j2\pi sm/S}$$

9 $${\overline{\mathbf{h}} }_{\vartheta }\left[m\right]={\sum }_{s=0}^{N-1}{\mathbf{h}}_{\vartheta }[s]{e}^{-j2\pi sm/S}$$

10 $$\overline{\mathbf{n} }\left[m\right]=\frac{1}{\sqrt{S}}{\sum }_{s=0}^{S-1}\mathbf{n}[s]{e}^{-j2\pi sm/S}$$

The system model in (6) can be written in vector shape as:

11 $$\underbrace {{\left[ {\begin{array}{*{20}c} {\begin{array}{*{20}c} {{\overline{\mathbf{z}}}\left[ 0 \right]} \\ \vdots \\ \end{array} } \\ {{\overline{\mathbf{z}}}\left[ {S - 1} \right]} \\ \end{array} } \right]}}_{{ = {\overline{\mathbf{z}}}}} = \underbrace {{\left[ {\begin{array}{*{20}c} {\begin{array}{*{20}c} {{\overline{\mathbf{h}}}_{\vartheta } \left[ 0 \right]} \\ \vdots \\ \end{array} } \\ {{\overline{\mathbf{h}}}_{\vartheta } \left[ {S - 1} \right]} \\ \end{array} } \right]}}_{{ = \overline{h}_{\vartheta } }} \odot \underbrace {{\left[ {\begin{array}{*{20}c} {\begin{array}{*{20}c} {{\overline{\mathbf{x}}}\left[ 0 \right]} \\ \vdots \\ \end{array} } \\ {{\overline{\mathbf{x}}}\left[ {S - 1} \right]} \\ \end{array} } \right]}}_{{ = {\overline{\mathbf{x}}}}} + \underbrace {{\left[ {\begin{array}{*{20}c} {\begin{array}{*{20}c} {{\overline{\mathbf{n}}}\left[ 0 \right]} \\ \vdots \\ \end{array} } \\ {{\overline{\mathbf{n}}}\left[ {S - 1} \right]} \\ \end{array} } \right]}}_{{ = {\overline{\mathbf{n}}}}}$$

where, $\odot$ denote the Hadamard (element-wise) product.

12 $${\overline{\mathbf{z}}} = {\overline{\mathbf{h}}}_{\vartheta } \odot {\overline{\mathbf{x}}} + {\overline{\mathbf{n}}},$$

where, Eq. (12) represents the relation between the input and output for one OFDM block and we can also notice that

13 $${\overline{\mathbf{h}} }_{\vartheta }={\varvec{F}}\left[\begin{array}{c}\begin{array}{c}{\mathbf{g}}_{0}^{\text{T}}{\mathbf{r}}_{\vartheta }\\ \vdots \end{array}\\ {\mathbf{g}}_{N - 1}^{\text{T}}{\mathbf{r}}_{\vartheta }\end{array}\right] ={\varvec{F}}{{\varvec{g}}}^{{\varvec{T}}}{\mathbf{r}}_{\vartheta }$$

where, ${{\varvec{h}}}_{u}$ containing all components of the uncontrollable channel, ${\varvec{g}}$ containing all components of the controllable channel and ${\varvec{F}}$ is $S \times N$ DFT matrix.

### IRS as reflection model

**Fig. 3:**
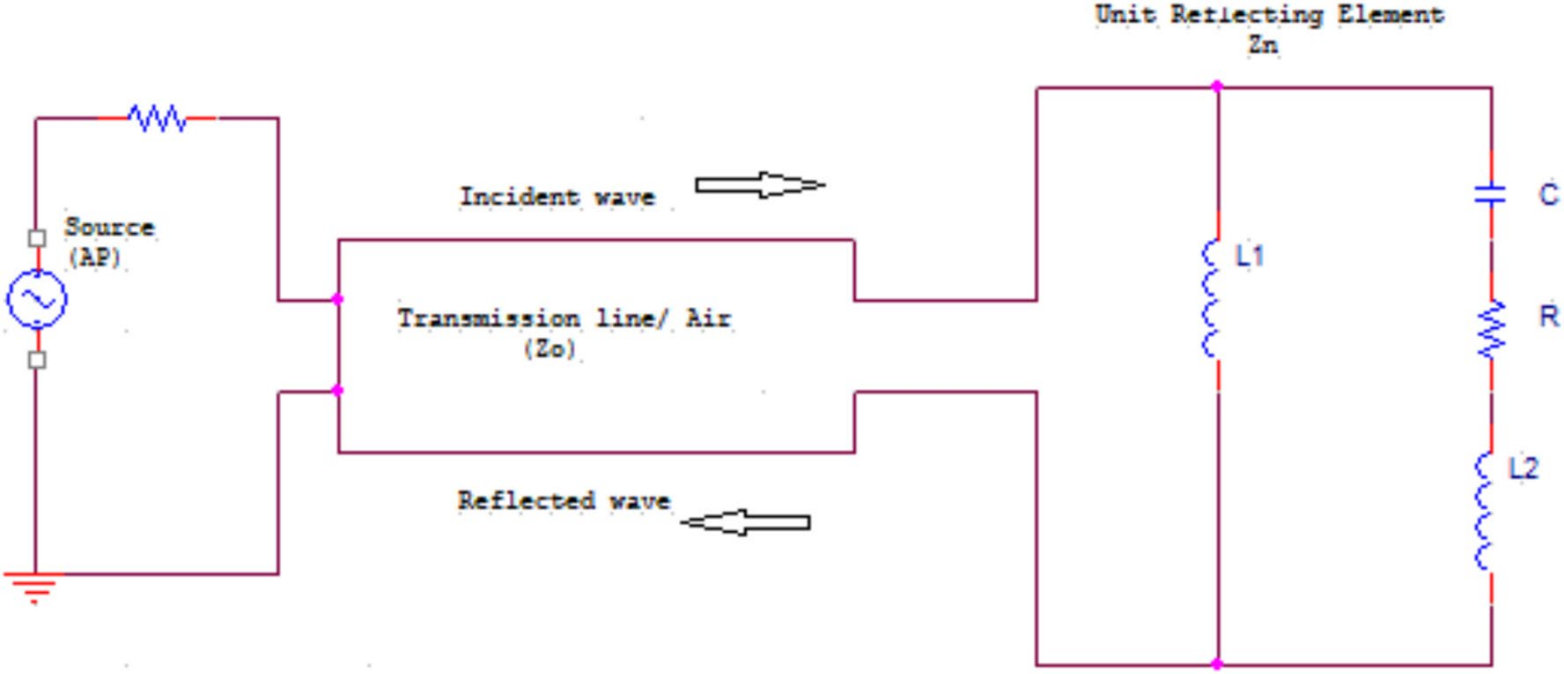
The equivalent circuit model of a unit reflecting element.

The IRS is typically formed as a (PCB) or printed circuit board, in which the distance between the reflecting components in a two-dimensional plane is equal. A unit reflecting element is constructed of a bottom layer with a full metal sheet and the PCB dielectric substrate top layer with a metal patch^14^. Furthermore, a semiconductor device is embedded into the metal patch on the uppermost layer. By controlling in the biasing voltage of this semiconductor the reflecting element impedance can be changed so that without varying the geometrical parameters the response of each element can be dynamically tuned^34^. Because the reflecting element physical length is smaller than the incident angle wavelength, the lumped circuit model can describe its response with neglect of the element geometry^35^. In reflecting elements, the metallic parts can be modeled as coils. When the current of high frequency flow on it generate magnetic field is nearly constant. The equivalent circuit model is illustrated in Fig. 3 for the $q$-th reflecting element.

The impedance of reflecting element is given by

14 $${Z}_{q}=\frac{j2\pi f{L}_{1}\left(j2\pi f{L}_{2}+\frac{1}{j2\pi fC}+R\right)}{j2\pi f{L}_{1}+j2\pi f{L}_{2}+\frac{1}{j2\pi fC}+R}$$

where, $C$ is the effective capacitance that can be controlled by a diode (${C}_{on}=0.5 \text{pF}, {C}_{off}=0.37 \text{pF})$,$R=1.5\Omega$ is the effective resistance, ${L}_{1}=3.5 \text{nH}$ is the bottom layer inductance, ${L}_{2}=1.8 \text{nH}$ is the top layer inductance and $f$ = 3.5 GHz is the frequency of the incident signal. Observe that $R$ determines the quantity of the power dispassion as a result of the losses in metals, dielectrics and semiconductor. The reflection coefficient of the reflecting element is determined as in^36^, which is given by

15 $$\Gamma = \frac{{Z}_{q}-{Z}_{o}}{{Z}_{q}+{Z}_{o}}$$

where, ${Z}_{o}$ is the free space impedance and ${Z}_{o}=377\Omega .$ So, whether a signal with a frequency $f$ arrives the IRS component, it will be reflected with amplitude of $\left|\Gamma \right|$ and shift in phase of arg(Γ). Under this IRS modeling approach, the reflection coefficients can be expressed as

16 $${\mathbf{r}}_{\vartheta }=\left[\begin{array}{c}\begin{array}{c}{\Gamma }_{1}\\ \vdots \end{array}\\ {\Gamma }_{q}\end{array}\right]$$

## Propagation environment and channel modeling

We assume that the AP is located at (30, −80, 0) in meters, while the IRS is located at the origin (as shown in Fig. 1) and the UEs are located in the azimuth plane within a square of size 15 m $\times$ 17 m and centered around (16.5, 1, 0). Let the uncontrollable path from the AP to any UE is blocked and this link is NLOS that is a main advantage for using an IRS to assist the communication^36^. Although the IRS is used for feature LOS path between the AP and most of the UEs, the case of NLOS path is considered in this paper where this case is occurred when the LOS path is blocked by obstacles near to the UE.

We consider all the scattering objects are positioned in the horizontal plane, so the array response vector in Eq. (2) will not depend on the elevation angle and can be simplified as follows:

17 $$A\left(\varphi ,\theta \right)=cos(\varphi )\left[\begin{array}{c}\begin{array}{c}{e}^{j0.8\pi sin(\varphi )i(1)}\\ \vdots \end{array}\\ {e}^{j0.8\pi sin(\varphi )i(Q)}\end{array}\right]\sqrt{D\left(\varphi ,\theta \right)} {\left[{e}^{j{K(\varphi ,\theta )}^{\text{T}}{U}_{1}}, . . . ,{e}^{j{K(\varphi ,\theta )}^{\text{T}}{U}_{Q}} \right]}^{\text{T}}$$

We can observe that the phase shifts only rely on the horizontal indices $i(q)$ that means when a wave hits from the azimuth angle $\varphi$, all the IRS elements in same column will receive similar signal copies.

The controllable path is described as^31^

18 $$\mathbf{g}=\sum_{l=1}^{{L}_{a}}\sum_{{\ell}=1}^{{L}_{b}}\sqrt{{\beta }_{a, l}{\beta }_{b,{\ell}}}{e}^{-j2\pi {f}_{c}\left( {\gamma }_{a,l}+{\gamma }_{b,{\ell}}\right)}(A({\varphi }_{a, l})\odot A({\varphi }_{b,{\ell}}))\times {\left[\begin{array}{c}\begin{array}{c}sinc(0+\mathbf{B}(\tau - {\gamma }_{a,l}-{\gamma }_{b,{\ell}}))\\ \vdots \end{array}\\ sinc(N-1+B(\tau - {\gamma }_{a,l}-{\gamma }_{b,{\ell}}))\end{array}\right]}^{T}$$

where, ${L}_{a}$ are the propagation paths in between the IRS and AP and ${L}_{b}$ is the propagation paths between the IRS and UE. ${\beta }_{a, l}\ge 0$ is the lth link pathloss between the AP and IRS, ${\gamma }_{a,l}$ is the propagation delay of this link and ${\varphi }_{a, l}$ is the incident angle which calculates the phase shifts of the IRS elements. While ${\beta }_{b,{\ell}}\ge 0$ is the lth link pathloss between the IRS and UE, ${\gamma }_{b,{\ell}}$ is the propagation delay of this link and ${\varphi }_{b,{\ell}}$ is the angle of departure which computes the phase shifts of the IRS elements. We assume 41 UEs and select 14 NLOS users at random among the first 40 UEs. The other users have LOS paths. There are S = 400 subcarriers and FIR filter with N = 20 taps.

In this setup, the optimized IRS configuration can be obtained by the Pilots transmission. All the signal $\overline{\mathbf{x} }$ elements in Eq. (12) are $\sqrt{{\text{P}}_{\mathbf{t}}/\text{B}}$ , where ${\text{P}}_{\mathbf{t}}/\text{B}$ is the transmitted power per symbol. When employing the IRS configuration ${\vartheta }_{c}$, the received signal can be written as

19 $${\overline{\text{z}} }_{c}=\sqrt{\frac{{\text{P}}_{t}}{\text{B}}}{\overline{\text{h}} }_{{\vartheta }_{c}}+{\overline{\mathbf{n}} }_{c}$$

where, the subscript c shows which configuration is considered. Then, we can determine the channel least square (LS) estimation as

20 $${\hat{\text{h}}}_{{\vartheta_{c} }} = \sqrt {\frac{{\text{B}}}{{{\mathbf{P}}_{{\mathbf{t}}} }}} {\overline{\text{z}}}_{c} = {\overline{\text{h}}}_{{\vartheta_{c} }} + \sqrt {\frac{{\text{B}}}{{{\mathbf{P}}_{{\mathbf{t}}} }}} {\overline{\mathbf{n}}}_{c}$$

To recognize optimized IRS configuration, pilots with C various configurations ${\vartheta }_{1}, \cdots , {\vartheta }_{c}$ could be transmitted, calculate the resulting channel by Eq. (20) and then select the one which provides the maximum rate. There are ${2}^{Q}$ likely configurations, C would have to be massive. So, a more effective approach is to use the pilot transmission to assess the channel components ${\varvec{g}}$. Where each element of IRS can only pick two states, the signal of the pilot will be depended on using the columns of Hadamard matrix ${{\varvec{H}}}_{Q}=\text{Q}\times \text{Q}$ with entries are + 1 or -1. By using the channel structure in Eq. (13), the received signal according to the pilot transmission can be described as

21 $${\text{Z = }}\sqrt {\frac{B}{{{\varvec{P}}_{{\varvec{t}}} }}} {\varvec{D}}\left( {{ }{\varvec{g}}^{T} \left[ {{\mathbf{r}}_{{\vartheta_{1} }} , \ldots ,{\mathbf{r}}_{{\vartheta_{C} }} } \right]} \right) + {\varvec{n}}{ } = \sqrt {\frac{B}{{{\varvec{P}}_{{\varvec{t}}} }}} {\varvec{D}}\left[ {{ }{\varvec{g}}^{T} } \right]\left[ {\begin{array}{*{20}c} {1, \ldots ,1} \\ {{\varvec{\Omega}}} \\ \end{array} } \right] + {\varvec{n}}$$

where, $\text{Z}=[{\overline{\mathbf{z}} }_{1},\cdots , {\overline{\mathbf{z}} }_{C}]$ includes all the received signals, ${\varvec{n}}=[{\overline{\mathbf{n}} }_{1},\cdots , {\overline{\mathbf{n}} }_{C}]$ is the noise matrix and ${\varvec{\Omega}} =[{\mathbf{r}}_{{\vartheta }_{1}},\cdots ,{\mathbf{r}}_{{\vartheta }_{C}}]$ contains all IRS configurations.

To obtains the received signals at a single UE, the pilot matrix ${\varvec{\Omega}}=\left[{{\varvec{H}}}_{Q}, -{{\varvec{H}}}_{Q},{\boldsymbol{ }{\varvec{H}}}_{Q,flip}, {-{\varvec{H}}}_{Q,flip}\right]$**;** where ${{\varvec{H}}}_{Q,flip}$ is the matrix that results from flipping each column upside-down, and $\text{C}=4\text{Q}$ received signal of OFDM blocks of the type in Eq. (19).

### Evaluation of spectral and energy efficiency for IRS-aided wireless communication system

According to the subcarriers in Eq. (6), the sum rate information can be expressed as^31^

22 $$\mathbf{R}=\frac{\text{B}}{S+N-1}\sum_{m=0}^{S-1}{{\varvec{l}}{\varvec{o}}{\varvec{g}}}_{2}(1+\frac{{\text{P}}_{\text{t}}{\left|{\overline{\mathbf{h}} }_{\vartheta }\left[m\right]\right|}^{2}}{{\text{BN}}_{o}})\boldsymbol{ }\boldsymbol{ }\boldsymbol{ }\boldsymbol{ }{\varvec{b}}{\varvec{i}}{\varvec{t}}/{\varvec{s}}$$

where, ${\text{P}}_{\text{t}}$ is the transmitted power and ${\text{N}}_{o}$ is the noise’s power spectral density.

23 $${\text{Spectral Effieciency}} = {\text{R}}/{\text{B}}\quad bit/s/{\text{Hz}}$$

24 $${\text{Energy}}\;{\text{Efficiency }} = { }\frac{{{\text{Total}}\;{\text{Data}}\;{\text{Rate}}\;{ }\left( {\text{ bps}} \right)}}{{{\text{Total}}\;{\text{Power}}\;{\text{Consumption}}\;\left( {\text{W}} \right)}}\quad {\text{bits}}/{\text{Joule}}$$

where, Total Data Rate is $\mathbf{R}$**,** total power consumption combines amplified transmit power and static power and is calculated according to the following equation:

25 $${\text{Total}}\;{\text{Power }}\;{\text{Consumption}} = \alpha *{\text{Transmitted}}\;{\text{power}} + {\text{P}}_{{{\text{fix}}}}$$

where, ${\varvec{\upalpha}}$ is the inverse of power amplifier efficiency $( v=0.8)$, ${\mathbf{P}}_{\mathbf{f}\mathbf{i}\mathbf{x}}$  includes circuit power for AP, users, and IRS elements. The circuit power of each user ${\text{p}}_{user}$=0.01 W, The circuit power of each AP ${\text{p}}_{AP}$=0.01 W and The circuit power of each IRS element ${\text{p}}_{IRS}$=0.001 W.

## Results and discussion

In this section, the discussion and presentation of all the technical problems illustrated in the preceding section of the current work will be examined with the results. First, the effect of components of IRS element on the performance of IRS-aided wireless communication system is introduced. Next, assessment performance of IRS-aided wireless communication system for single user using key metrics such as SNR, data rate, spectral efficiency and energy efficiency is studied. Finally, the same metrics are applied to assess the performance of IRS-aided wireless communication system in a multi-user scenario.

### The effect of components of IRS element on the performance of IRS-aided wireless communication system

The $\text{C}, R, {L}_{1}, {L}_{2}\text{ and} f$ are fundamental considerations in IRS hardware design, as they directly influence the performance of reconfigurable unit cells. In the following subsections, the impact of them on the reflected signals will be studied.

#### Dependance of the reflected signals on both of the resistance and effective capacitance of the IRS elements

**Fig. 4:**
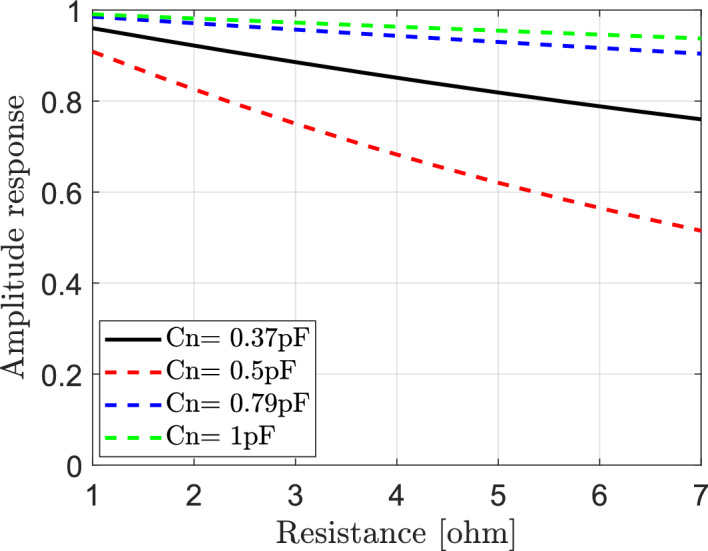
Dependence of amplitude response of reflection coefficient on the effective resistance Rn of IRS elements at different values of the effective capacitance Cn.

**Fig. 5:**
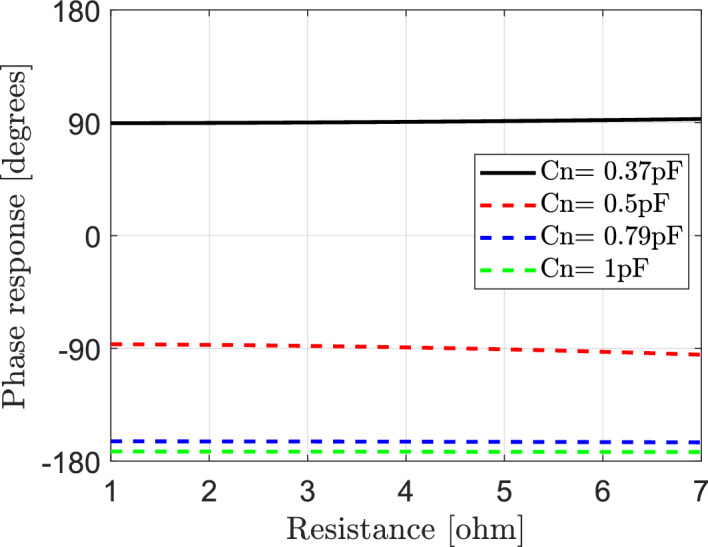
Dependence of phase response of reflection coefficient on the effective resistance Rn of IRS elements at different values of the effective capacitance Cn.

It may be important to study the effect of variation for both of effective resistance and capacitance of elements of IRS on reflected signals in IRS-aided wireless communication networks. The reflection coefficient is calculated according to Eq. (15). Each element of IRS is assumed to has bottom layer inductance ${L}_{1}=3.5{\text{ nH}}$, top layer inductance ${L}_{2}=1.2{\text{ nH}}$ and the frequency of the incident signal is $f=3.5$ GHz. The variation of both of effective resistance Rn and capacitance Cn can impact the reflection coefficient’s phase and amplitude, which can affect the constructive or destructive interference patterns of the reflected signals and also affect the overall validation of wireless communication system assisted by IRS. The relation between the effective resistance Rn and the magnitude of reflection coefficient is presented in Fig. 4 for various values of effective capacitance of elements of IRS. It should be noted that, at $1\Omega \le \text{Rn}\le 7\Omega$ and $0.37 \text{pF}\le \text{Cn}\le 1 \text{pF}$ increasing of the effective resistance at different values of effective capacitance leads to decrease in the amplitude response and consequently decrease the magnitude of reflected signal. In Fig. 5 the relation between the effective resistance and phase of reflection coefficient is also presented at various values of effective capacitance of elements of IRS. It is observed that the phase response is not be affected with increasing of the effective resistance but the variation of the capacitance affect the phase response.

#### Effect of variation of the effective capacitance and the bottom layer inductance (${\mathbf{L}}_{1}$) of elements of IRS on the reflected signals

**Fig. 6:**
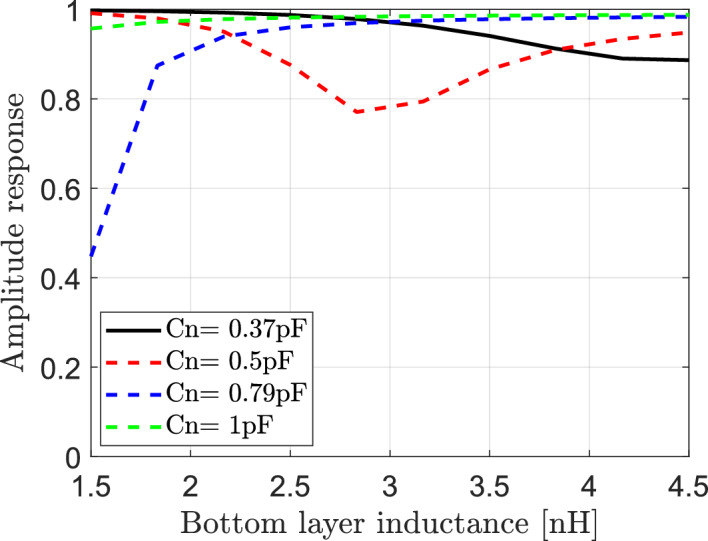
Dependence of amplitude response of reflection coefficient on the bottom layer inductance ${\text{L}}_{1}$ of IRS elements at different values of effective capacitance Cn.

**Fig. 7:**
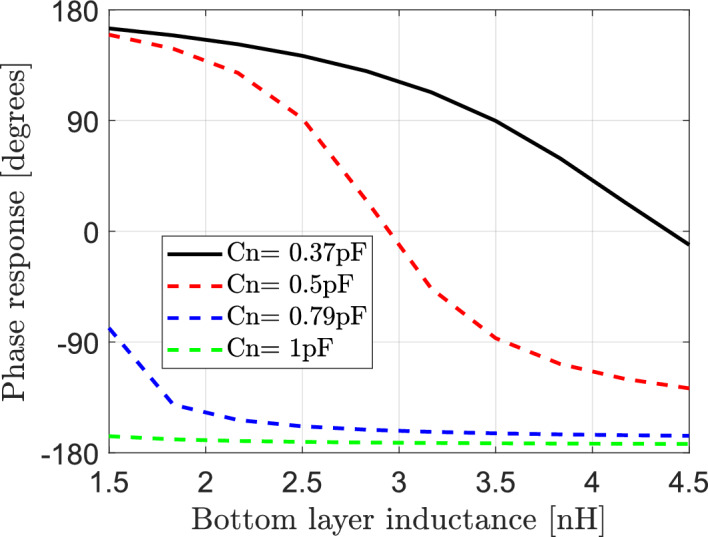
Dependence of phase response of reflection coefficient on the bottom layer inductance ${\text{L}}_{1}$ of IRS elements at different values of effective capacitance Cn.

The dependence of reflected signals in IRS-aided wireless communication system on variation for both of effective capacitance Cn and bottom layer inductance ${\text{L}}_{1}$ of elements of IRS is studied in this section. From Eq. (15) of the reflection coefficient, it is assumed that each element of IRS has the effective resistance $R = 1.5\Omega$, top layer inductance ${L}_{2}=1.2{\text{ nH}}$ and the frequency of the incident signal is $f=3.5$ GHz. The variation of both of effective capacitance Cn and the bottom layer inductance ${\text{L}}_{1}$ can impact the amplitude and phase of the reflection coefficient. In Fig. 6, the relation between the bottom layer inductance ${\text{L}}_{1}$ and the magnitude of reflection coefficient is presented for different values of the effective capacitance. It is clear that the relation between the amplitude response and the bottom layer inductance is not constant and depends on values of effective capacitance Cn. At Cn = 0.37pF, the increasing of values of the bottom layer inductance results in decreasing the amplitude response and consequently decrease the reflected signal. While for Cn = 0.79, the increasing of the bottom layer inductance values results in increase the amplitude response and consequently increase the reflected signal. In Fig. 7 the relation between the bottom layer inductance and phase of reflection coefficient is also presented for various values of effective capacitance of elements of IRS. It is clear that the effective capacitance plays a crucial role in controlling of phase shift of reflected signals where the phase response changes from $-\pi to \pi$ over a range of bottom layer inductance values. By adjusting values of the capacitance and bottom layer inductance, the IRS can manipulate the phase of the incident signal and steer it towards the desired direction. This enables beamforming and beam steering capability, allowing for signal enhancement in specific directions.

#### Dependence of the reflected signals on both of the effective capacitance and the top layer inductance (${\mathbf{L}}_{2}$) of IRS elements

**Fig. 8:**
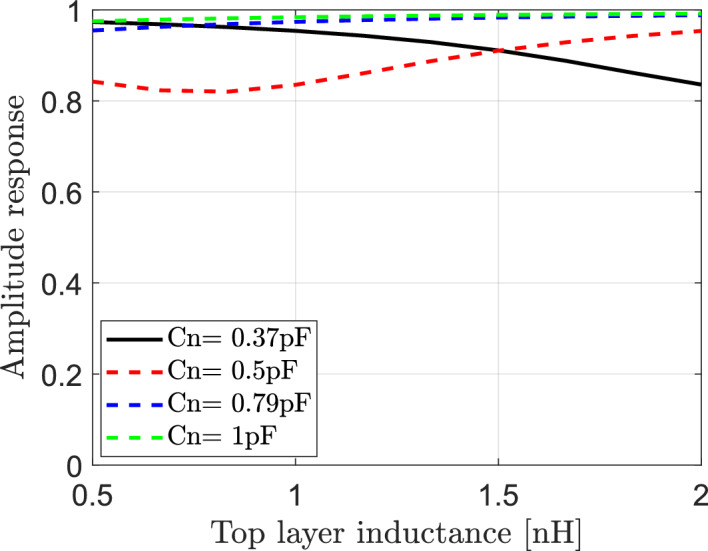
Dependence of amplitude response of reflection coefficient on the top layer inductance (${\text{L}}_{2}$) of IRS elements for different values of the effective capacitance Cn.

**Fig. 9:**
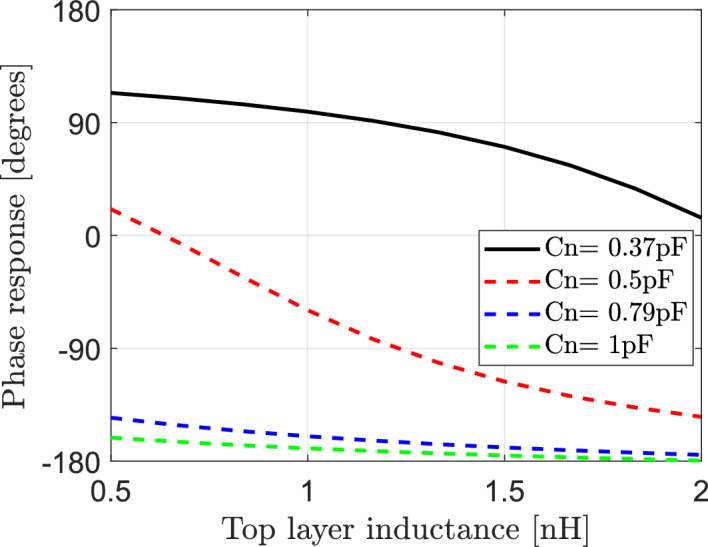
Dependence of phase response of reflection coefficient on the top layer inductance (${\text{L}}_{2}$) of IRS elements for different values of the effective capacitance Cn.

The effect of both of effective capacitance Cn and bottom layer inductance (${\text{L}}_{2}$) of elements of IRS on reflected signals in IRS-aided wireless communication networks is investigated in this section. The reflection coefficient is calculated according to Eq. (15). Each element of IRS is assumed to has effective resistance $R = 1.5\Omega$, bottom layer inductance ${L}_{1}=3.5\text{nH}$ and the frequency of the incident signal is $f=3.5$ GHz. The variation for both of the top layer inductance ${L}_{2}$ and the effective capacitance Cn can impact the amplitude and phase of the reflection coefficient. The relation between the amplitude response and the top layer inductance is calculated and plotted for different values of $\text{Cn}$ as shown in Fig. 8. It is clear that the relation between the amplitude response and ${L}_{2}$ is not constant and depends on the effective capacitance. For Cn = 0.37pF, the increasing of ${L}_{2}$ results in decrease the amplitude response and consequently decrease the reflected signal. For Cn = 0.5pF, the increasing of ${L}_{2}$ results in increase the amplitude response and consequently increase the reflected signal. While for Cn = 0.79pF and Cn = 1pF, the amplitude response is constant at any value for top layer inductance. In Fig. 9 the relation between the top layer inductance and phase of reflection coefficient is also presented for various values of $\text{Cn}$ of elements of IRS. It is clear that the top layer inductance plays a crucial role in controlling of phase shift of reflected signals where the phase response changes from $-\pi to \pi$ at different values of the effective capacitance.

#### Dependence of the reflected signals on the frequency of the incident signal and the IRS elements

**Fig. 10:**
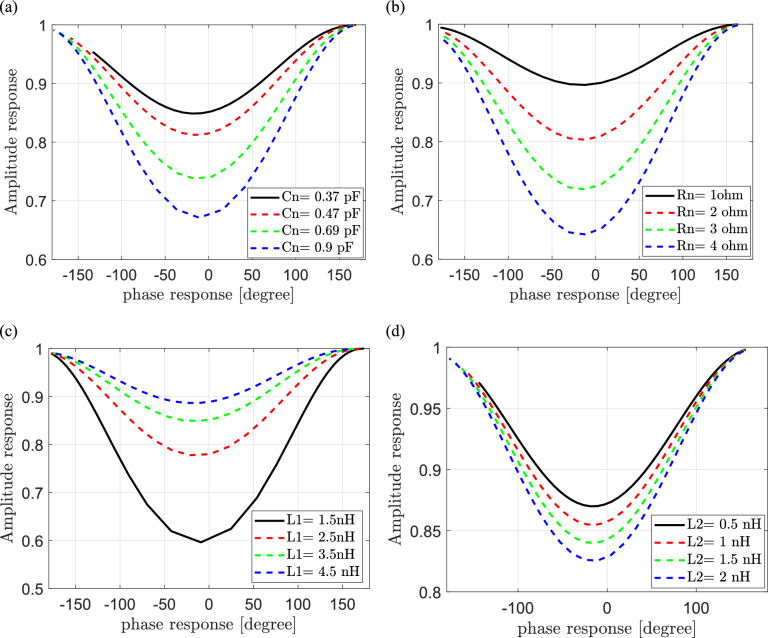
Dependence of phase and amplitude response on the frequency of the incident signal at different values of (**a**) the effective capacitance Cn, (**b**) the effective resistance Rn, (**c**) the bottom layer inductance ${\text{L}}_{1} and$ (**d**) the top layer inductance ${\text{L}}_{2}$ of components of IRS elements.

The effect of frequency of the incident signal on reflected signal in IRS-supported wireless communication networks is investigated in this section. The reflection coefficient is calculated according to Eq. (15). The relation between the amplitude response and the phase response is calculated and plotted over a range of frequencies (where $1< f \le 7)$ at different values for each component of IRS elements as shown in Fig. 10a–d. In Fig. 10a, the amplitude response is plotted versus the phase response at different values of the effective capacitance Cn. It is show that lower values of capacitance results in the better values of amplitude response over phase response ranges from −$\pi \text{to} \pi .$ In Fig. 10b, the amplitude response is plotted versus the phase response at different values of the effective resistance Rn. It is also clear that lower values of resistance results in the better values of amplitude response over phase response ranges from −$\pi \text{to} \pi .$ In Fig. 10c, the amplitude response is plotted versus the phase response at different values of the bottom layer inductance ${\text{L}}_{1}.$ It is observed that higher values of this inductance results in the better values of amplitude response over phase response ranges from −$\pi \text{to} \pi .$ In Fig. 10d, the amplitude response is plotted versus the phase response at different values of the top layer inductance ${\text{L}}_{2}.$ It is show that lower values of this inductance results in the better values of amplitude response over phase response ranges from -$\pi \text{to} \pi .$ One can conclude that using of lower values of each the effective capacitance, resistance, top layer inductance and using of higher values of bottom layer inductance in form of the IRS elements increase the values of reflection coefficients and consequently improve the performance of IRS-assisted wireless communication systems.

### Performance assessment of IRS-aided 6G wireless communication system for single user

In this section, the dependence of the performance of the wireless communication networks that employ the IRS on the location of transmitting AP according to IRS position, the size of IRS, the transmitted power and bandwidth for single user will be studied. The received power, the signal to noise ratio (SNR), and spectral efficiency are investigated and utilized as measures for system performance evaluation at the strongest and weakest configuration for IRS elements (where the strongest and weakest configurations refer to the specific arrangements and phase shift assignments of the IRS elements that lead to either the best or worst performance. The strongest configuration is the one that maximizes the desired performance metric. The weakest configuration is the one that minimizes the desired performance metric). In the end of this section, comparison between the spectral efficiency of system with IRS and without IRS is presented at different values of the transmitted power and bandwidth.

#### Dependence of IRS-aided wireless communication system on the location of transmitting AP according to IRS position

**Fig. 11:**
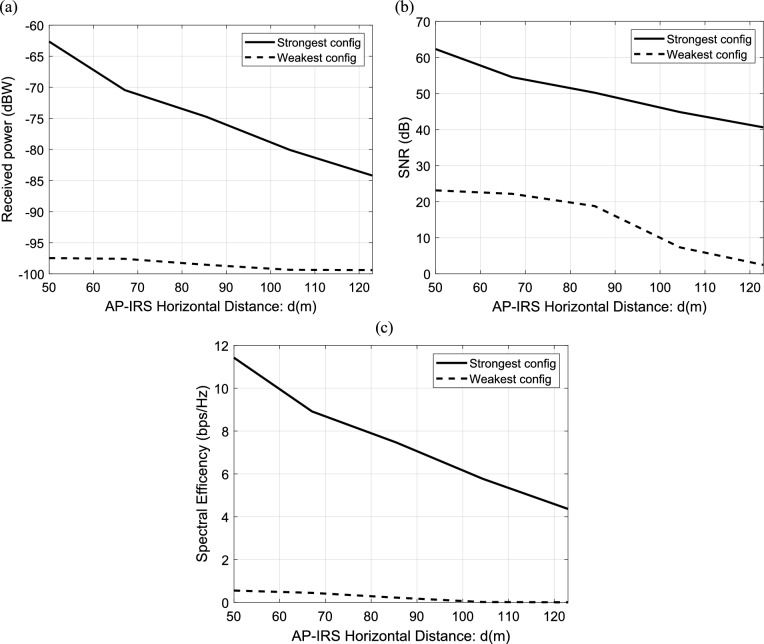
Dependence of (**a**) the received power, (**b**) the corresponding SNR and (**c**) the spectral efficiency in IRS-aided wireless communication system on AP-IRS horizontal distance, f = 3.5 GHz, the IRS dimensions are ${Q}_{x} \times {Q}_{y}=32\times 32$ element, R = 1.5 $\Omega$, ${L}_{1}=3.5\text{nH}, {L}_{2}=1.8{\text{ nH}}, {C}_{on}=0.5\text{ pF}, {C}_{off}=0.37\text{ pF},\text{ B}=10{\text{ MHz}},{S = 400\text{ subcarriers}, N=20\text{ taps},\text{P}}_{t}=1\text{W },{\text{N}}_{o}=3.16e-20\frac{\hbox{W}}{\hbox{Hz}}$ .

The effect of location of transmitting AP according to IRS position on the performance of IRS-aided wireless communication system is investigated in this section. The received power in dB is calculated and plotted versus AP-IRS horizontal distance in meter for the strongest and weakest configuration of IRS elements as shown in Fig. 11a. As expected, the received power decreases with increasing of AP-IRS horizontal distance. In Fig. 11b, the relation between the SNR in dB and the horizontal distance from AP to IRS is calculated and plotted for the strongest and weakest configuration. It is clear that the SNR decreases with increase AP-IRS horizontal distance. In Fig. 11c, the data rate in bps/Hz (spectral efficiency) is calculated and plotted versus AP-IRS horizontal distance in meter for the strongest and weakest configuration of IRS elements. It should be noticed that the increasing in AP-IRS horizontal distance leads to decrease the spectral efficiency and this affect the performance of the system. Also, it is clear that the strongest configuration results in the best received power, the best SNR and, hence, the best spectral efficiency.

#### Dependence of IRS-aided wireless communication system on the transmitted power

**Fig. 12:**
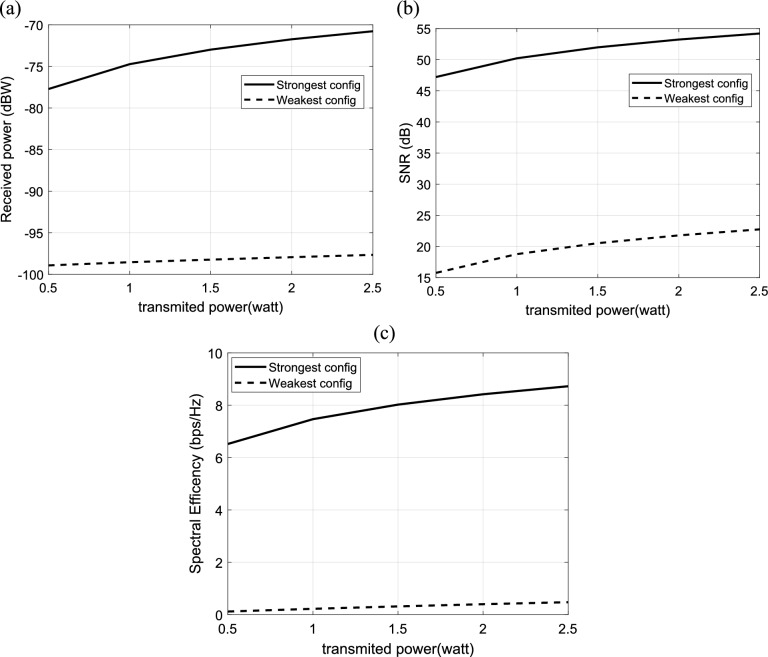
Dependence of (**a**) the received power, (**b**) the corresponding SNR and (**c**) the spectral efficiency in IRS-aided wireless communication system on transmitted Power, f = 3.5 GHz, the IRS dimensions are ${Q}_{x} \times {Q}_{y}=32\times 32$ element, d = 85.44 m, R = 1.5 $\Omega$, ${L}_{1}=3.5\text{nH}, {L}_{2}=1.8{\text{ nH}}, {C}_{on}=0.5\text{ pF}, {C}_{off}=0.37\text{ pF},S = 400\text{ subcarriers}, N=20\text{ taps},\text{ B}=10{\text{ MHz}},{\text{N}}_{o}=3.16e-20\frac{\hbox{W}}{\hbox{Hz}}$.

The effect of the transmitted power on the performance of IRS-aided wireless communication system is investigated in this section. The received power at the users is calculated and plotted versus the transmitted power for the strongest and weakest configuration of IRS elements as shown in Fig. 12a. The received power increases linearly with the transmitted power, as expected. The relation between SNR and the transmitted power is calculated and plotted in Fig. 12b. It is clear that the SNR increases with increasing of the transmitted power. The variation of the transmitted power and the corresponding spectral efficiency with the strongest and weakest configuration of IRS elements is presented in Fig. 12c. It should be noted that the increasing in the transmitted power results in decrease the spectral efficiency. Also, it is clear that the strongest configuration results in the best received power, the best SNR and, hence the best spectral efficiency.

#### Dependence of IRS-aided wireless communication system on the size of IRS

**Fig. 13:**
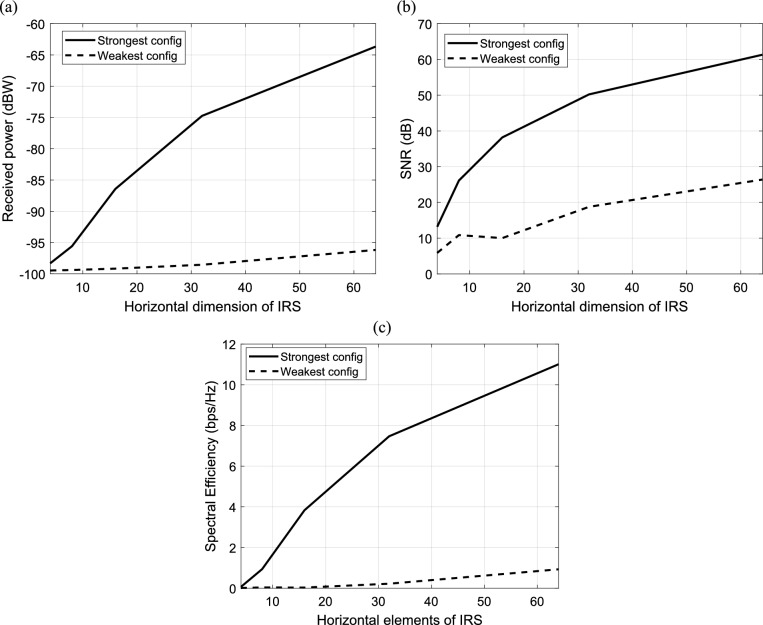
Dependence of (**a**) the received power, (**b**) the corresponding SNR and (**c**) the spectral efficiency in IRS-aided wireless communication system on horizontal dimension of IRS, f = 3.5 GHz, d = 85.44 m, R = 1.5 $\Omega$, ${L}_{1}=3.5\text{nH}, {L}_{2}=1.8{\text{ nH}}, {C}_{on}=0.5\text{ pF}, {C}_{off}=0.37\text{ pF},\text{ B}=10{\text{ MHz}},S = 400\text{ subcarriers}, N=20\text{ taps},{\text{N}}_{o}=3.16e-20\frac{\hbox{W}}{\hbox{Hz}}$ , ${\text{P}}_{t}=1\text{W}$.

The effect of the size of IRS on performance of IRS-aided wireless communication system is investigated in this section. The received power is calculated and plotted versus the horizontal dimension of IRS (where the horizontal dimension is equal to the vertical dimension) for the strongest and weakest configuration of IRS elements as shown in Fig. 13a. For the strongest configuration, the received power increases linearly with the horizontal dimension of IRS; while for the weakest configuration, the received power is nearly constant for any size of IRS. In Fig. 13b, the SNR is calculated and plotted. In Fig. 13c, spectral efficiency is calculated and plotted versus the horizontal dimension of IRS for the strongest and weakest configuration of IRS elements. It should be noticed that the increasing in the horizontal dimension of IRS leads to increase the spectral efficiency and this upgrade the performance of the system. It is clear that the strongest configuration results in the best received power, the best SNR and, hence the best spectral efficiency.

#### Dependence of IRS-aided wireless communication system on the bandwidth

**Fig. 14:**
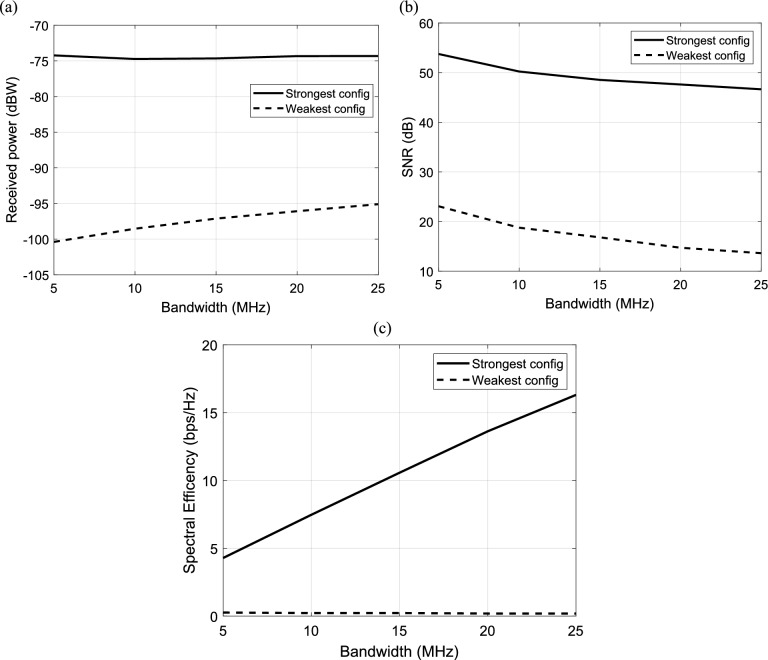
Dependence of (**a**) the received power, (**b**) the corresponding SNR and (**c**) the spectral efficiency in IRS-aided wireless communication system on bandwidth, f = 3.5 GHz, the IRS dimensions are ${Q}_{x} \times {Q}_{y}=32\times 32$ element, d = 85.44 m, R = 1.5 $\Omega$, ${L}_{1}=3.5\text{nH}, {L}_{2}=1.8{\text{ nH}}, {C}_{on}=0.5\text{ pF}, {C}_{off}=0.37\text{ pF}, {\text{P}}_{t}=1\text{W},{\text{ N}}_{o}=3.16e-20\frac{\hbox{W}}{\hbox{Hz}}$ .

The effect of the bandwidth on the performance of IRS-aided wireless communication system is studied in this section. The relation between the received power and bandwidth is calculated and plotted for the strongest and weakest configuration of IRS elements as illustrated in Fig. 14a. The received power increases linearly with the bandwidth at the weakest configuration, while at the strongest configuration, the received power is nearly constant at any value for the bandwidth. In Fig. 14b, the SNR is calculated and plotted versus the bandwidth. It is clear that the SNR decreases linearly with the increasing of bandwidth at both of the strongest and weakest configuration. Also, it is noted that the strongest configuration results in the best SNR. The variation of the bandwidth and the corresponding spectral efficiency with the strongest and weakest configuration of IRS elements is presented in Fig. 14c. It should be noted that the increasing in the bandwidth results in increase the spectral efficiency and consequently improve the performance of the system.

#### Analytical performance comparison between the spectral efficiency of system with IRS and without IRS

**Fig. 15:**
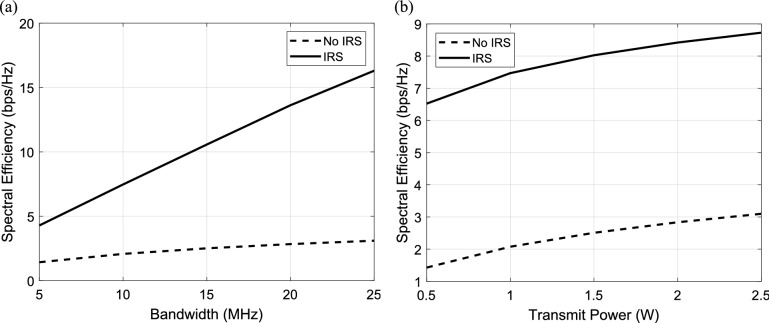
Comparison between the spectral efficiency in IRS-aided wireless communication system and wireless communication system without IRS at different values of (**a**) The Bandwidth and (**b**) The transmit power.

We examine the effectiveness of the IRS and the basic communication scenario without the assist of IRS in this section. In Fig. 15a the relation between bandwidth and data rate is calculated and plotted for the system with IRS and without IRS. It is clear that, the spectral efficiency of the system in case of IRS is higher than without IRS. At bandwidth = 5 MHz, the system with IRS achieves nearly 3 times the spectral efficiency of the system without IRS. Also, it is clear that the difference between the spectral efficiency in both cases (IRS and without IRS) increases with increasing the value of bandwidth. At bandwidth = 25 MHz, the system with IRS achieves nearly 14 times the spectral efficiency of the system without IRS. In Fig. 15b the dependence of spectral efficiency on the transmitted power is computed and plotted for the system with IRS and without IRS. It is clear that at any value of transmitted power the spectral efficiency for the system with IRS is better than the system without IRS. At transmitted power = 0.5 Watt, the system with IRS achieves nearly 5 times the spectral efficiency of the system without IRS. It is clear that, the improvement can be increased with increasing the values of the transmitted power. At transmitted power = 2.5 Watt, the system with IRS achieves nearly 5.7 times the spectral efficiency of the system without IRS. One can conclude that IRS can improve the spectral efficiency of the system and consequently improve the performance of the system.

### Assessment of performance of IRS-aided 6G wireless communication system in a multi-user scenario

**Fig. 16:**
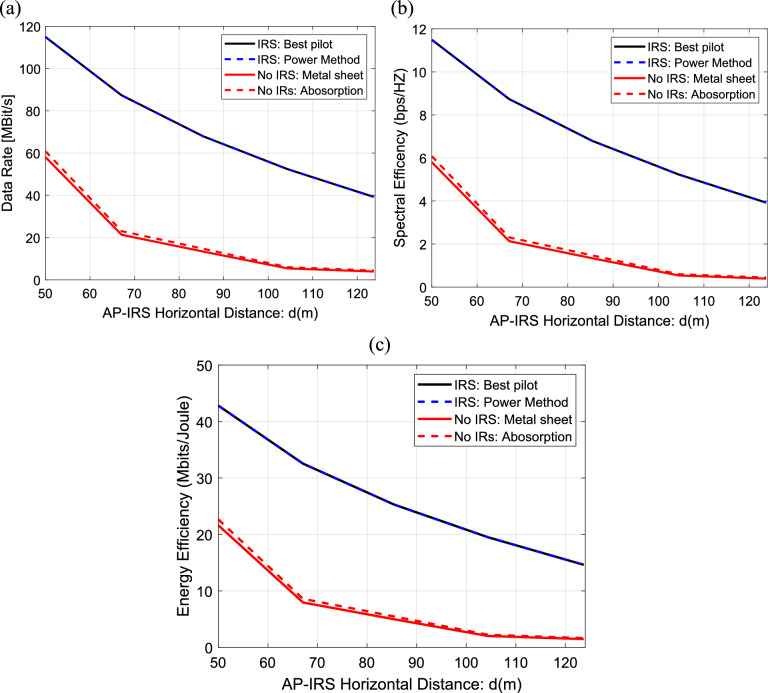
Dependence of (**a**) The data rate, (**b**) The spectral efficiency and (**c**) The energy efficiency on the AP-IRS horizontal distance in IRS-aided wireless communication system, f = 3.5 GHz, the IRS dimensions are ${Q}_{x} \times {Q}_{y}=32\times 32$ element, R = 1.5 $\Omega$, ${L}_{1}=3.5\text{nH}, {L}_{2}=1.8{\text{ nH}}, {C}_{on}=0.5\text{ pF}, {C}_{off}=0.37\text{ pF},S = 400\text{ subcarriers}, N=20\text{ taps}, {\text{P}}_{t}=1\text{W},{\text{ N}}_{o}=3.16e-20\frac{\hbox{W}}{\hbox{Hz}}$ , B = 10 MH.

In this section, we will investigate the effect of the AP-IRS horizontal distance, the size of IRS, the transmitted power and bandwidth on the performance of IRS-aided wireless communication system in a multi-user scenario. The data rate (where the data rate is averaged over random realizations of the multipath components), the spectral and energy efficiency are used as a metrics for the performance. In this paper, we will use the proposed power method in^31^ to obtain the best data rates and consequently, the best spectral and energy efficiency. In this method, the goal is to maximize the received signal power. The results of this method will be compared with uniform metal surface method, IRS with best pilot method and with the case of no IRS. The data rate, the spectral efficiency and the energy efficiency are calculated and plotted versus the AP-IRS horizontal distance for the previous four methods as shown in Fig. 16a–c. As expected, the data rate, the spectral efficiency and the energy efficiency decrease linearly with increasing of the AP-IRS horizontal distance. It is clear that both of the power method and the best pilot method result in the best data rate, spectral and energy efficiency values among the four methods for all the distance values. Also, it is clear that, in the subplots of the three metrics, their results in case of using a uniform metal surface (metal sheet) is nearly equal to their results in case of no IRS (absorption).

**Fig. 17:**
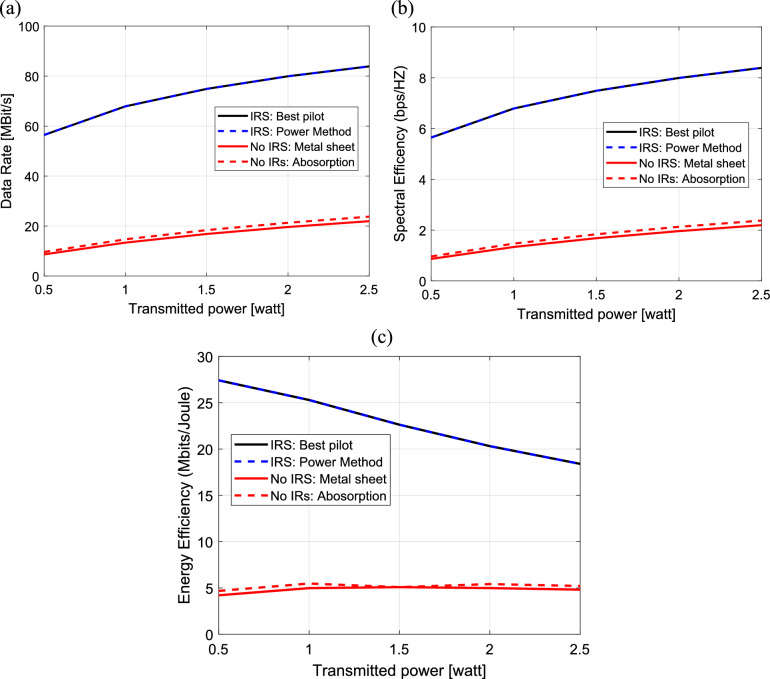
Dependence of (**a**) The data rate, (**b**) The spectral efficiency and (**c**) The energy efficiency on the transmitted power in IRS-aided wireless communication system, f = 3.5 GHz, the IRS dimensions are ${Q}_{x} \times {Q}_{y}=32\times 32$ element, d = 85.44 m, R = 1.5 $\Omega$, ${L}_{1}=3.5\text{nH}, {L}_{2}=1.8{\text{ nH}}, {C}_{on}=0.5\text{ pF}, {C}_{off}=0.37\text{ pF},S = 400\text{ subcarriers}, N=20\text{ taps},{\text{ N}}_{o}=3.16e-20\frac{\hbox{W}}{\hbox{Hz}}$ , B = 10 MH.

The relation between the data rate, spectral and energy efficiency is computed and plotted against the transmitted power for the same methods that are mentioned before in the previous paragraph as shown in Fig. 17a–c. In Fig. 17a, the data rate increases linearly with the transmitted power for all four methods. In Fig. 17b, the spectral efficiency also increases linearly with the transmitted power for all four methods. However, in Fig. 17c, the energy efficiency decreases as transmitted power increases across all the four methods. Also, it is clear that, the power method and the best pilot method result in the best data rate, spectral and energy efficiency for all the transmitted power levels.

**Fig. 18:**
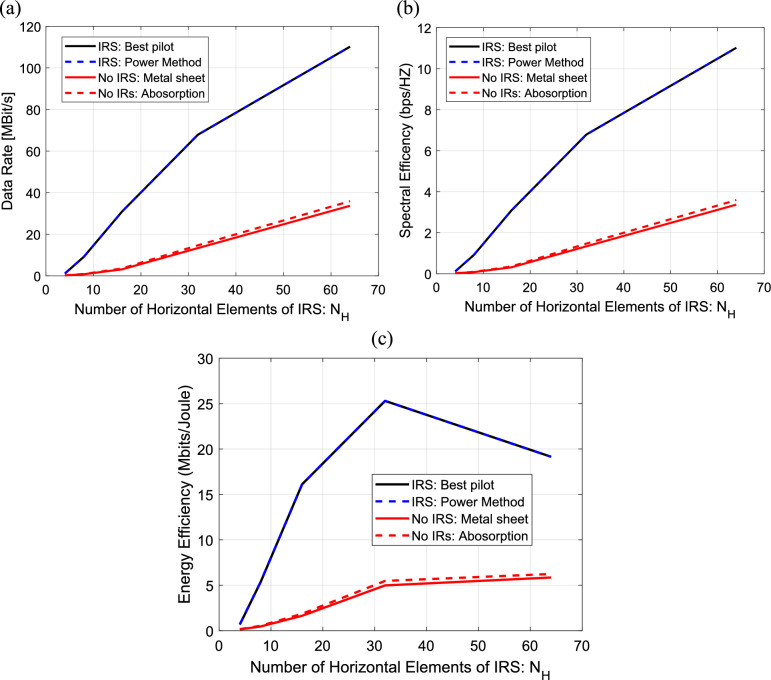
Dependence of (**a**) The data rate, (**b**) The spectral efficiency and (**c**) The energy efficiency on the number of horizontal elements of IRS in IRS-aided wireless communication system, f = 3.5 GHz, the IRS dimensions are element, d = 85.44 m, R = 1.5 $\Omega$, ${L}_{1}=3.5\text{nH}, {L}_{2}=1.8{\text{ nH}}, {C}_{on}=0.5\text{ pF}, {C}_{off}=0.37\text{ pF},S = 400\text{ subcarriers}, N=20\text{ taps}, {\text{P}}_{t}=1\text{W},{\text{ N}}_{o}=3.16e-20\frac{\hbox{W}}{\hbox{Hz}}$ , B = 10 MH.

In Fig. 18a–c, the data rate, spectral and energy efficiency are calculated and plotted versus the number of horizontal elements of IRS for power method, uniform metal surface method, IRS with best pilot method and with the case of no IRS. The data rate increases linearly with the number of IRS elements for all four methods as shown in Fig. 18a. In Fig. 18b, the spectral efficiency also increases linearly with the number of horizontal elements of IRS for all four methods. In Fig. 18c, the energy efficiency increases linearly with the number of horizontal elements of IRS till ${\text{N}}_{\text{H}}=32$ elements. At ${\text{N}}_{\text{H}}>32 ,$ the energy efficiency decreases linearly with increasing number of IRS elements for all four methods. It is clear that the power method and the best pilot method result in the best data rate and spectral efficiency for all the numbers of IRS elements but, the large increasing in IRS elements has bad effect on the energy efficiency.

**Fig. 19:**
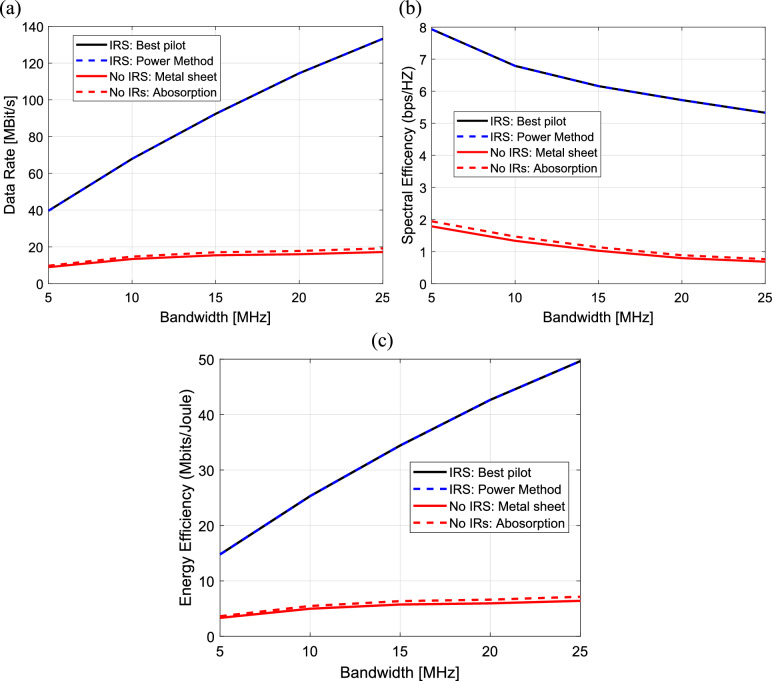
Dependence of (**a**) The data rate, (**b**) The spectral efficiency and (**c**) The energy efficiency on the bandwidth in IRS-aided wireless communication system, f = 3.5 GHz, the IRS dimensions are ${Q}_{x} \times {Q}_{y}=32\times 32$ element, d = 85.44 m, R = 1.5 $\Omega$, ${L}_{1}=3.5\text{nH}, {L}_{2}=1.8{\text{ nH}}, {C}_{on}=0.5\text{ pF}, {C}_{off}=0.37\text{ pF},S = 400\text{ subcarriers}, N=20\text{ taps}, {\text{P}}_{t}=1\text{W},{\text{ N}}_{o}=3.16e-20\frac{\hbox{W}}{\hbox{Hz}}$ .

The data rate, spectral and energy efficiency are computed and plotted against the bandwidth for all four methods as shown in Fig. 19a–c. The data rate increases linearly with the bandwidth for all four methods as shown in Fig. 19a. In Fig. 19c, the energy efficiency also increases linearly with bandwidth for all four methods. However, In Fig. 19b, the spectral efficiency decreases linearly with increasing of bandwidth for all four methods. It is clear that the power method and the best pilot method result in the best data rate, energy and spectral efficiency for all the bandwidth levels.

Based on the preceding findings, it can be concluded that an increase in the AP-IRS horizontal distance results in a reduction of the data rate, spectral and energy efficiency. While an increase in the transmitted power leads to improve of the data rate, spectral efficiency and a reduction of the energy efficiency. Also, an increase in the number of IRS elements results in improve of the data rate, spectral efficiency and the energy efficiency but, a large increasing in number of IRS elements (at ${\text{N}}_{\text{H}}>32)$ leads to a reduction in energy efficiency. However, an increase in the bandwidth results in improve of the data rate, energy efficiency and a reduction of the spectral efficiency.

## Conclusion

In this paper, the performance of IRS-aided wireless communication system when the direct link between the Base BS and receiver is obstructed has been investigated by using the pilot transmission and channel least square method in calculation of the received signal. We firstly study the effect of components of IRS element on the performance of IRS-aided wireless communication system. From the presented numerical results, it has been shown that each of the effective capacitance $C$, bottom layer inductance ${L}_{1}$, top layer inductance ${L}_{2}$, the effective resistance R of IRS elements and the frequency of the incident signal have a major effect on the reflection coefficient of the reflected signal and hence, on the performance of IRS-aided wireless communication system. Then, assessment performance of IRS-aided wireless communication system for single user is presented. The received power, SNR, and the spectral efficiency were estimated and used as metrics for the system performance. It has been shown that the received power, SNR and the spectral efficiency are increased with increasing the size of IRS and transmitted power for both of the strongest and weakest configuration. Also, it has been shown that the received power, SNR and the spectral efficiency are decreased with increasing the AP-IRS horizontal distance. Higher degree of bandwidth improves the received power and the spectral efficiency whereas it reduces the SNR. Analytical performance comparison between the spectral efficiency of system with IRS and without IRS at different values of transmitted power and bandwidth is introduced. It is obvious that, at bandwidth = 5 MHz, the system with IRS achieves nearly 3 times the spectral efficiency of the system without IRS and at bandwidth = 25 MHz, the system with IRS achieves nearly 14 times the spectral efficiency of the system without IRS. Also, at transmitted power = 0.5 Watt, the system with IRS achieves nearly 5 times the spectral efficiency of the system without IRS and at transmitted power = 2.5 Watt, the system with IRS achieves nearly 5.7 times the spectral efficiency of the system without IRS. So, The IRS can be used and aided in wireless communication. Finally, assessment of performance of IRS-aided 6G wireless communication system in a multi-user scenario is presented. The data rate, spectral and energy efficiency were evaluated by using four different methods (power method, uniform metal surface method, IRS with best pilot method and with the case of no IRS). It has been shown that the received power method and the best pilot method give the best data rate, spectral and energy efficiency at any value for each of the transmitted power, the bandwidth, the size of IRS and AP-IRS horizontal distance and consequently, improve the performance of IRS- aided 6G wireless communication network.

## Data Availability

The datasets used and/or analysed during the current study available from the corresponding author on reasonable request.
